# Supplementary figures and images for: miR-1-5p targets TGF-βR1 and is suppressed in the hypertrophying hearts of rats with pulmonary arterial hypertension
Source: PLoS One. 2020 Feb 28;15(2):e0229409. doi: 10.1371/journal.pone.0229409 (PMC7048278; doi:10.1371/journal.pone.0229409)

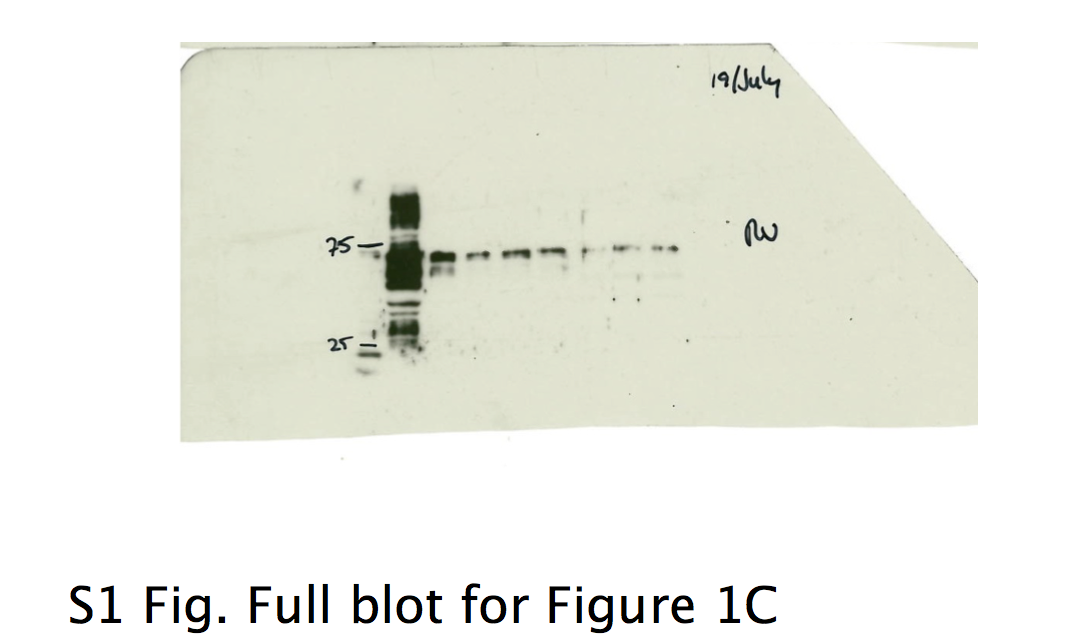

Supplement: S1 Fig — (TIFF) [file pone.0229409.s001.tiff]

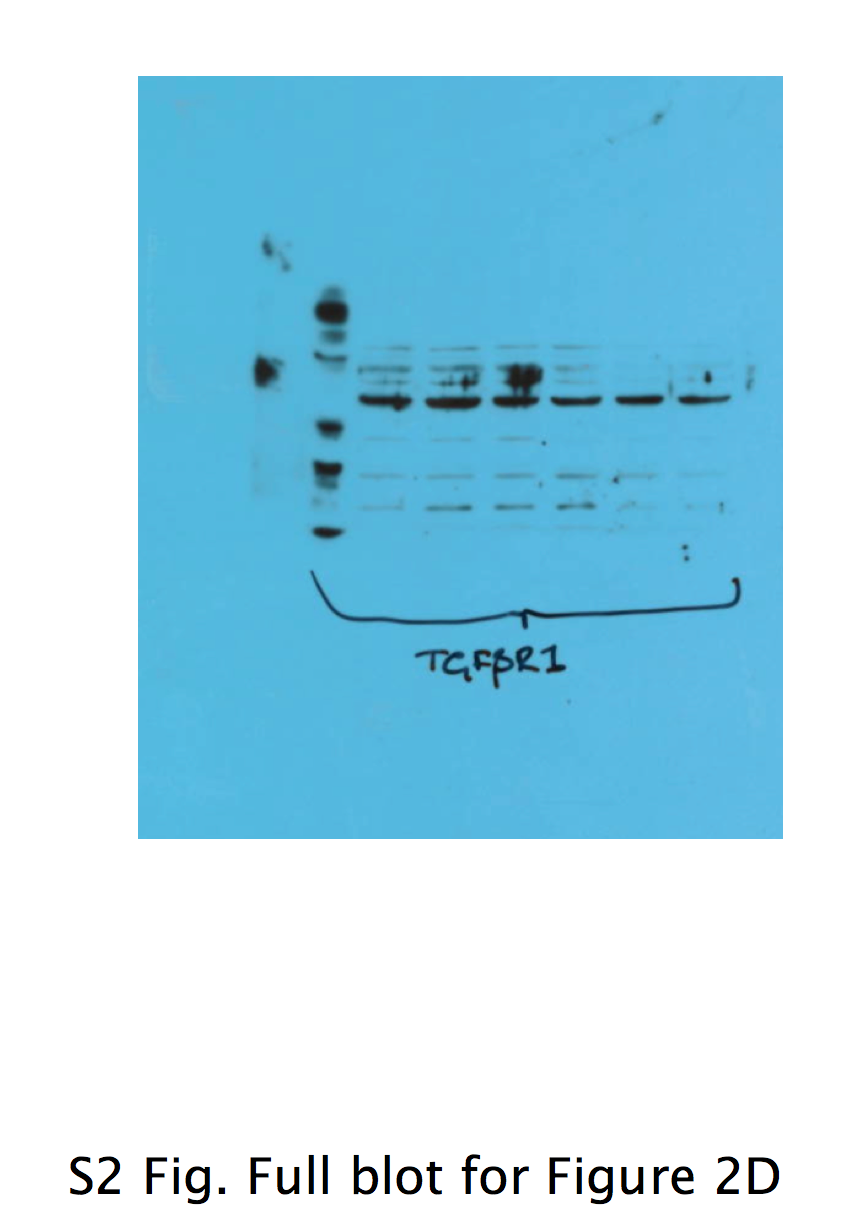

Supplement: S2 Fig — (TIFF) [file pone.0229409.s002.tiff]
